# Selenium-Dependent Antioxidant Enzymes: Actions and Properties of Selenoproteins

**DOI:** 10.3390/antiox7050066

**Published:** 2018-05-14

**Authors:** Evangelos Zoidis, Isidoros Seremelis, Nikolaos Kontopoulos, Georgios P. Danezis

**Affiliations:** 1 Department of Nutritional Physiology and Feeding, Faculty of Animal Science and Aquaculture, Agricultural University of Athens, 75 Iera Odos, 11855 Athens, Greece; 2 Chemistry Laboratory, Department of Food Science and Human Nutrition, Agricultural University of Athens, 75 Iera Odos, 11855 Athens, Greece; iseremelis@gmail.com (I.S.); stud315044@aua.gr (N.K.); gdanezis@aua.gr (G.P.D.)

**Keywords:** antioxidant system, reactive oxygen species (ROS), selenium, selenocysteine, selenomethionine, selenoproteins

## Abstract

Unlike other essential trace elements that interact with proteins in the form of cofactors, selenium (Se) becomes co-translationally incorporated into the polypeptide chain as part of 21st naturally occurring amino acid, selenocysteine (Sec), encoded by the UGA codon. Any protein that includes Sec in its polypeptide chain is defined as selenoprotein. Members of the selenoproteins family exert various functions and their synthesis depends on specific cofactors and on dietary Se. The Se intake in productive animals such as chickens affect nutrient utilization, production performances, antioxidative status and responses of the immune system. Although several functions of selenoproteins are unknown, many disorders are related to alterations in selenoprotein expression or activity. Selenium insufficiency and polymorphisms or mutations in selenoproteins’ genes and synthesis cofactors are involved in the pathophysiology of many diseases, including cardiovascular disorders, immune dysfunctions, cancer, muscle and bone disorders, endocrine functions and neurological disorders. Finally, heavy metal poisoning decreases mRNA levels of selenoproteins and increases mRNA levels of inflammatory factors, underlying the antagonistic effect of Se. This review is an update on Se dependent antioxidant enzymes, presenting the current state of the art and is focusing on results obtained mainly in chicken.

## 1. Introduction

Selenium (Se) is an essential trace element which is co-translationally incorporated into the polypeptide chain as part of the amino acid selenocysteine (Sec). Proteins that include Sec in their polypeptide chain are defined as selenoproteins and exist in all lineages of life (archaea, bacteria and eukarya) [1,2,3,4,5,6], however, some organisms do not have the ability to synthesize selenoproteins [7]. Selenoproteins have pivotal significance for optimal human and animal health mainly due to their antioxidant activity [8]. They have anti-inflammatory, chemo preventive and antiviral properties and are related with the improvement of immune responses [9,10]. The health-related properties of selenoproteins include among others protection against cancer [11,12], proper thyroid function [13] and protection against cardiovascular [14] and muscle disorders; however, the role of selenium supplementation in diseases of the thyroid gland is still subject to discussion [15,16,17,18,19,20,21].

There are 25 genes encoding for selenoproteins [22,23]. Selenoprotein family, in human, includes the following members: glutathione peroxidases (GPX1–GPX4 and GPX6), thioredoxin reductases (TXNRD1–2), thioredoxin-glutathione reductase (TXNRD3), iodothyronine deiodinases (DIO1–3), selenophosphate synthetase (SEPHS2), 15-kDa selenoprotein (SELENOF), selenoprotein H (SELENOH), selenoprotein I (SELENOI), selenoprotein K (SELENOK), selenoprotein M (SELENOM), selenoprotein N (SELENON), selenoprotein O (SELENOO), selenoprotein P (SELENOP), methionine sulfoxide reductase B1 (MSRB1), selenoprotein S (SELENOS), selenoprotein T (SELENOT), selenoprotein V (SELENOV) and selenoprotein W (SELENOW) [24,25]. Low dietary Se may result in selenoproteins deficiency leading to such human diseases as Keshan disease [26,27,28], Kashin-Beck disease [29], myxedematous endemic cretinism [30] and male infertility [31].

According to the Office of Dietary Supplements (ODS) of the National Institutes of Health (NIH, Bethesda, MD, USA), Recommended Daily Allowance (RDA) of Se is 55 μg/day for men of 31–50 years old up to 70 μg/day for women on lactation. Even though globally dietary Se intake is usually below 55 μg/day [8], European countries such as Greece [32], Poland [33], UK [34] and Spain [35] have an average Se intake below 40 μg/day and this appears to be the situation also for other parts of the world. For example, it has been estimated that the average Se intake for adult men and women in Argentina is 32 and 24 μg/day, respectively [36]. Further, a recent study carried out from German, Austrian and Swiss nutrition societies resulted in values of 70 μg/day for men and 60 μg/day for women using the reference body weights [37]. To help meeting the RDA, consumers could benefit from the use of Se-enriched functional foods. Production technology of Se-enriched eggs, milk and meat has been developed and successfully introduced. Actually, Se-enriched eggs can be obtained in more than 25 countries worldwide [36,38].

## 2. Synthesis of Selenoproteins from Dietary Selenium

Selenocysteine and selenomethionine (SeMet) are similar to cysteine and methionine except that the sulfur (S) atom is substituted by Se [39]. Yeast and plants do not incorporate Sec and, therefore, do not possess selenoproteins [40]. However, in a recent study, the analysis of cranberry mitochondrial genome revealed the presence of two copies of selenocysteine insertion sequence element (SECIS) and tRNA-Sec [41]. Plants absorb Se from the soil in the form of selenate or selenite and synthesize SeMet [42], which implies that in natural feed ingredients Se can be primarily found in the form of SeMet [43]. Vertebrates get dietary Se as SeMet and other Se-amino acids, including Sec and its methylated forms, depending on their contents in feed/food components. Moreover, presently, farm animal feeds are broadly supplemented with inorganic Se such as sodium-selenate and selenite as well as with organic sources of Se such as selenized yeast. There are major differences in the metabolism and absorption of these forms of Se. For example, sodium selenite is passively absorbed in the intestine and more efficiently in the ileum segment [44], while SeMet is actively absorbed and thus requires a transport mechanism to actually shift the molecules through the enterocyte cell membrane, by all segments of the intestine [45]. Absorption of Se compounds into cells is considered to occur via anion transporters [46,47,48]. Irrespective of the form that Se is received from the diet, it has to be metabolized into Sec to be incorporated into selenoproteins.

The incorporation of Se as Sec into a selenoprotein requires a specific mechanism to decode the UGA codon in mRNA, which normally operates in translation termination [49]. Initially, the oxidized forms of inorganic Se undergo reductive metabolism yielding hydrogen selenide (H_2_Se) which is converted to Sec [50]. Organic sources of Se can also produce H_2_Se. Selenomethionine can be trans-selenated into Sec, similarly to the trans-sulfuration pathway for methionine to cysteine, before lysis by β-lyase to H_2_Se. Hydrogen selenide must be first transformed to Sec, which means that H_2_Se has to be metabolized into selenophosphate after catalysis by the SEPHS2 [50]. Selenophosphate reacts with the tRNA specific for serine Ser-tRNASec via the enzyme Sec-tRNA synthase to give Sec bound tRNA (Sec-tRNASec), from which Sec is incorporated into selenoproteins by the Sec specific UGA codon [42,51,52,53,54]. Specific mutations in the coding sequence of the Sec-tRNA play a key role in the expression of particular selenoproteins but also in the progression and symptomatology of some diseases [55]. This incorporation of Sec into selenoproteins occurs when the mRNA contains a distinct hairpin mRNA sequence downstream of the UGA codon in its 3′-untranslated region (3′-UTR) called Sec insertion sequence (SECIS) [56]. Sec insertion sequence prevents the termination of translation by competing for release factors that would otherwise lead to disassembly of the mRNA-ribosomal complex [53,57,58,59,60]. Biosynthesis of selenoproteins requires binding of SBP2 (SECIS-binding protein 2) to the SECIS element and recruitment of the Sec tRNA-specific elongation factor (EFsec), connected to tRNASec [61]. Namely, SECIS recruits SBP2 and binds the tRNASec-loaded EFSec, while several additional proteins bind to SECIS [62,63,64].

Selenium may also efficiently control UGA-Sec codon translation [65,66], regulate levels of Sec tRNASer(Sec) and control the ratio of the methylated and unmethylated Sec tRNA(Ser)Sec isoforms [67,68,69,70]. The methylated isoform is translationally active and Se-induced tRNA methylation is a mechanism of selenoprotein synthesis regulation. The methylated isoform has been shown to govern the synthesis of selenoproteins involved in the oxidative stress response such as GPX1 and GPX3, whereas the unmethylated form controls synthesis of housekeeping selenoproteins i.e., TXNRD1 and TXNRD3 [71].

It seems that all selenoproteins of eukaryotes require a form of the SECIS element for recoding UGA to the Sec codon [61]. However, there may be a SBP2 independent binding affinity for the Sec-tRNA. This must be clarified by further research [72]. Most of the selenoproteins can be classified into two groups according to the position of the Sec in the polypeptide chain. In the first group, Sec is located on the N-terminal while in the second group Sec is present on the C-terminal position of the function domain [73]. The first group includes all glutathione peroxidases and iodothyronine deiodinases, SEPHS2, SELENOF, SELENOH, SELENOM, SELENON, SELENOP, SELENOT, SELENOV, and SELENOW, while the second one includes thioredoxin reductases, SELENOS, MSRB1, SELENOO, SELENOI and SELENOK [50].

The first selenoenzyme identified—glutathione peroxidase (GPX)—catalyzes the oxidation of cytosolic glutathione (GSH) by H_2_O_2_ (Table 1). GPX1 was discovered in erythrocytes [74], where it removes H_2_O_2_ formed by dissociation of oxyhemoglobin into O_2_ and methemoglobin [75]. The GPX1 gene codes for both the cytosolic and mitochondrial forms of the enzyme, which exist as a tetramer of four identical subunits, each containing a single Sec residue.

## 3. Selenoproteins and the Antioxidant System

The lack of balance between generation of reactive oxygen/nitrogen species (ROS/RNS) and the action of protective antioxidant systems is defined as oxidative stress [76,77]. In normal conditions, a balance between generation of free radicals and antioxidant protection exists in the cell. However, increased ROS/RNS generation or impairment of the antioxidant protective ability leads to oxidative stress. Generation of ROS by a reduced coupling of oxidation and phosphorylation in the inner mitochondrial membrane results in an enhanced electron leakage and overproduction of superoxide radicals (Table 1).

Many pollutants, such as heavy metals (e.g., Cd and Pb), certain drugs and various radiations (ultra violet and γ rays), can induce high production of free radicals. When the generation of ROS surpasses the capacity of the antioxidant system to neutralize them, extensive peroxidation of lipids occurs and causes damage to the unsaturated lipids of the cellular membrane. As a result, the integrity of the cell is disturbed. In addition, disturbances of natural antioxidant and/or metallothionein (MT) levels may cause peroxidation of lipids, which may result in ROS attacking and cleaving of double bonds. It is generally accepted that the susceptibility of polyunsaturated fatty acids (PUFA) to peroxidation corresponds to the number of double bounds in these molecules. In fact, arachidonic acid (AA, 20:4n-6) and docosahexaenoic acid (DHA, 22:6n-3) constitute the primary substrates of the peroxidation in the membrane. The same PUFA are mainly responsible for maintenance of physiologically crucial properties of the membrane including permeability and fluidity. In other words, both function and structure of biological membranes are compromised through lipid peroxidation [78]. This is correlated with reduced absorption of various nutrients and causes an imbalance of inorganic elements, amino acids, vitamins and other nutrients in the organism [79]. Thus, oxidative stress decreases productive and reproductive performances of organisms [80]. About 100,000 ROS hit rat cellular DNA each day [81,82,83]. DNA damage may occur via oxidation, methylation, deamination and depurination. Reactive oxygen species interaction with DNA is very complex and includes strand break, base modification, DNA-protein cross links and lesions in chromatin and sugars [84,85,86]. Organisms have evolutionary developed specific protective antioxidant mechanisms to defend against ROS/RNS. Consequently, it is the presence of natural antioxidants in living organisms that enables them to survive in an oxygen-rich environment [87].

Other major targets of two-electron oxidants and radicals in biological systems are proteins due to their rapid rates constants and abundance for reaction [88,89]. Protein oxidation by free radicals can occur in the protein backbone or at amino acid side chains [90]. Peroxides (protein peroxidation) and peroxyl radicals are formed under oxygen presence. They can oxidize both proteins and other targets. Protein modifications by oxidation, can include unstable intermediates formation, loss of the parent amino acid residue and also generation of stable products. Protein oxidation is linked with multiple human pathologies and aging [88,91,92,93,94]. Selenoproteins as wide range antioxidants also protect cell from damages caused by cellular proteins oxidation [95]. Further, selenium-containing compounds such as selenides (RSeR’), selenols (RSeH) and diselenides (RSeSeR’) react with peroxynitrite under raised rate constants [96].

The antioxidant system is mainly comprised of water-soluble antioxidants (uric acid, ascorbic acid, and taurine); natural fat-soluble antioxidants (carotenoids, vitamins A and E, and ubiquinones); antioxidant enzymes; the thioredoxin system (thioredoxin/thioredoxin peroxidase/thioredoxin reductases (TXNRDs)); and the thiol redox system (GSH/GSH reductase/glutaredoxin/GPXs) [97]. For maximum protection, the antioxidant system is located in various organelles, subcellular compartments as well as the extracellular space.

In vertebrates, GPXs have a single redox center and as redox-active residue the selenocystein. In contrast, non-vertebrate GPXs replace peroxidatic Sec by a cysteine (peroxidatic Cys) and work with a second redox center that contains a resolving cysteine [98].

Among the components of the thioredoxin and thiol redox systems, several enzymes contain Sec residues that are essential for enzyme function [99]. Nonetheless, most, if not all, of the currently known selenoproteins are also found as Cys-containing non-selenoprotein orthologs in other organisms, wherefore any potentially unique properties of selenoproteins are yet a matter of debate. The chemical rate enhancement obtained with Sec can have other consequences than those arising from a low redox potential of some Cys-dependent proteins, typically aiming at maintaining redox equilibria. Another unique aspect of Sec compared to Cys seems to be its efficient potency to support one-electron transfer reactions, which, however, has not yet been unequivocally shown as a Sec-dependent step during the natural catalysis of any known selenoprotein enzyme [100].

The antioxidant system of the cell includes three major levels of defense [101]. The first level (prevention) prevents the formation of free radical. It inactivates catalysts or removes precursors of free radicals and includes three antioxidant enzymes namely GPX, superoxide dismutase (SOD) and catalase (CAT) and metal-binding proteins such as transferrin, lactoferrin, haptoglobin, hemopexin, metallothionein, ceruloplasmin, ferritin, albumin and myoglobin. Thus, metal sequestration is an important part of extracellular antioxidant defense [80,102,103]. This first level of antioxidant defense is insufficient to fully impede generation of free radical in the cell. As a result, some radicals escape, initiating peroxidation of lipids and causing protein and DNA damages. However, lipids peroxidation can only be stopped by GPX4 and no other selenoprotein. Thus, a second level (interception) of antioxidant defense exists. It consists of chain-breaking antioxidants (vitamins A and E, ascorbic acid, uric acid, carotenoids, and ubiquinol) as well as other antioxidants. Thioredoxin and thiol redox systems play also an essential role in this second level of defense. Thioredoxin reductases use nicotinamide adenine dinucleotide phosphate to reduce oxidized thioredoxin and its homologs, which regulate many redox signaling events. Vitamin E cannot do the whole work in preventing peroxidation of lipids by scavenging free radicals and forming hydroperoxides [103]. Together with Se, they act in tandem; and even very high doses of dietary vitamin E cannot replace Se, which is indispensable to complete the second part of antioxidant defense. Thus, Se as an integral part of selenoproteins contribute both to the first and the second level of antioxidant defense [97].

Elemental Se and more often its compounds have been successfully used as stoichiometric reagents and catalysts for oxidation of different organic substrates. Selenium(IV) oxide, areneseleninic acids and their anhydrides are widely used as stoichiometric oxidants or as oxygen-transfer agents for oxygen donors, particularly hydrogen peroxide and tert-butyl hydroperoxide. The oxidation mechanisms depend on the substrate and oxidant or catalyst used. The electrophilic center localized on the selenium atom or the nucleophilic center localized on the oxygen atom of the selenahydroperoxide group are involved in the reaction mechanism [104].

While ROS are largely involved in causing cell damage, they also possess significant roles in several physiological aspects such as regulation of protein phosphorylation, intracellular signal transduction, regulation of the cytosolic Ca concentration, regulation of gene expression, intracellular killing of bacteria by neutrophils and macrophages and activation of certain transcription factors, such as nuclear factor-kappa B (NF-κB) and the activator protein 1 (AP-1) family factors [105,106,107,108,109,110,111]. Free radical reactions are part of normal human metabolism and can be induced by environmental sources [87,112].

Glutathione is the most abundant non-protein thiol in mammalian and avian cells, providing them with their reducing milieu [113,114]. Cellular GSH has an essential role in various biological processes including: synthesis of proteins and DNA [115,116], cell proliferation and growth [117], regulation of apoptosis and cancer growth [118], immune regulation (T cell growth and metabolic reprogramming) [119,120], xenobiotic metabolism, transport of amino acids and redox-sensitive signal transduction [121]. Moreover, GSH thiolic group may directly react with several ROS including hydroxyl radicals, hydroperoxides, alkoxyl radicals and superoxide anion (Table 1) [80,122,123]. Most importantly, GSH acts as scavenger of free radical, especially efficient against the hydroxyl radical, since there are no enzymatic defenses against this radical species [124]. Generally, reduced concentration of GSH in tissues is connected with increased peroxidation of lipids [101,125]. Moreover, in stress conditions, GSH prevents the loss of vitamin E and protein thiols [126] and acts as a key modulator of cell signaling [127]. Humans as well as animals can synthesize GSH. In this respect, eight GPX members have been discovered [97,128]. Apart from their specific functions, all of them exert antioxidant tasks, protecting cells against oxidation damage from free radicals and ROS [129].

Selenoproteins have the unique ability to trap H_2_O_2_ and use the resulting selenenic acid to form specific disulfide bonds in the presence of 10–20 mM GSH and are thus well suited for controlling the formation of disulfide bonds with specific Cys residues. Because of the rapid oxidation kinetics of selenols, H_2_O_2_ in the cytosol reacts completely with Sec-containing proteins to produce Sec selenenic acid before it can react with other potential targets. Thus, rapid consumption of H_2_O_2_ by selenoproteins acts to limit the duration of H_2_O_2_ signals and the distance over which those signals are transmitted by diffusion [130].

Nevertheless, even this second level of cell antioxidant defense (interception) is unable to hinder the deteriorating effects of ROS/RNS on DNA and proteins. In this case, the third level of antioxidant defense depends on systems that repair damaged molecules or eliminate them [131]. This level of defense consists of proteolytic (peptidases or proteases), lipolytic (lipases) and other enzymes (proteinases, phospholipases, nucleases, DNA repair enzymes, polymerases, ligases and diverse transferases). However, the enzymes of the third level of antioxidant defense cannot achieve total removal or repair of damaged DNA molecules and this may result to arrest of cell cycle and cell death [132]. In fact, the process of apoptosis (programmed cell death in multicellular organisms) is involved in maintenance of the genetic integrity by eliminating genetically altered cells [78].

## 4. Effects of Selenium Supplementation in Chicken

As already stated, the major role of Se is the protection against free radical detrimental effects via the action of selenoproteins. Many studies have shown that Se/vitamin E dietary deficiencies are related to presumed oxidative cell damage. It has been shown that selenoproteins impede nutritional muscular dystrophy (NMD) initiation in chicks via metabolism of peroxides and redox regulation mechanisms [133]. In addition, dietary Se deficiency has a negative impact in the mRNA expression of 23 selenoproteins in broiler immune organs (thymus, spleen and bursa). Additionally, at Se super-nutritional status, most selenoproteins were upregulated in chicken kidney [134,135,136]. Moreover, 24 selenoproteins and associated transcription factors were influenced by Se intake in chickens’ pancreas. Selenium contributions to chicken development and regulation of blood sugar were associated with pancreatic redox homeostasis via a modulated selenotranscriptome [137]. Selenoprotein genes in spleen and thymus were down-regulated, inflammation-related genes were up-regulated due to supranutritional dietary Se status. Further, chickens’ growth performances were depressed. Over high ambient temperature, Se enriched probiotics reduced the effects of Se deficiency on heart lesion. In addition, in chicken heart, upregulation of mRNA expression of GPX1 and GPX4 and downregulation of heat shock protein genes (HSP90, HSP70 and HSP60) were induced with Se enriched probiotics [138] and, similarly, in broilers exposed at high ambient temperature, prebiotics enriched with Se improved meat quality [139]. Notably, investigations have reported the protective role of selenoprotein W against H_2_O_2_ which means that an association of selenoprotein W mRNA expression, oxidative stress and metabolic status may exist. Moreover, selenoprotein W expression could be regulated by metabolic factors and Cd in fish [140].

Improved action of Se containing antioxidant enzymes, for instance TXNRD and GPX, decreased free radical-mediated peroxidation and regeneration of GSH was observed due to Se supplementation [141]. Thus, Se levels above nutritional requirements may be capable of maintaining optimal TXNRD and GPX activities and detoxifying Cd [142,143,144].

Many studies highlight the effectiveness of Se in preventing heavy metal poisoning. A recent study revealed Se-Cr (VI) interaction and other trace elements accumulation in chicken serum and brain. The results demonstrated that Se supplementation decreased accumulation of Cr both in chicken brain and serum and this interaction regulated the correlation and contents of Ca, Cu, Mn, Fe, Zn, and Mg in these tissues [145]. Another study investigated the effects of Se on the toxicity of Pb and the expressions of selenoproteins mRNA in chicken cartilage tissue. The results showed that reduced expression of GPX4, GPX2, GPX1, DIO1–2, TXNRD2–3, selenoprotein U, selenoprotein I, Sepx1, selenoprotein O, selenoprotein M, selenoprotein K, selenoprotein W, selenoprotein 15, Sepn1, selenoprotein T, and selenoprotein S in the meniscus cartilage, triggered by Pb exposure, might be alleviated by Se. In addition, in cartilage of sword fish, the reduction in the mRNA expression levels of GPX2–4, TXNRD1–2, DIO2–3, selenoprotein H, selenoprotein I, SEPHS2, MSRB1, selenoprotein W, selenoprotein K, selenoprotein M, selenoprotein O, selenoprotein 15, selenoprotein T, Sepn1 and selenoprotein Pb due to Pb exposure could be mitigated by Se [146].

Lead toxicity mitigative effect by Se has been investigated via expression analysis of 25 selenoproteins and 5 HSPs. The results indicated that Se declined the increase of HSPs mRNA expression and enhanced the diminished mRNA expression levels of all selenoproteins caused by Pb in chicken testes, pointing that HSP70 could be an indicator of Pb poisoning in this tissue [147]. Moreover, Pb exposure increased Pb content in serum, activated the NF-κB pathway, and increased the expression of selenoproteins in chicken neutrophils. Selenium supplements could reduce Pb concentration in serum, had a mitigative effect on the activation of the NF-κB pathway and further enhanced the upward trend of selenoproteins expression induced by Pb exposure [148]. Further studies investigated the antagonistic effect of Se on Pb-induced inflammation injury and the results showed that Pb poisoning enhanced mRNA levels of inflammation, reduced mRNA levels of selenoproteins, and induced histological modifications in chicken hearts. Except Pb, toxicity was also induced by Cd. Thus, studies aimed to evaluate potential cytoprotective mechanisms of Se and to examine the actions of Se in mitigating Cd induced toxicity in chicken heart. It seems that Cd can cause serious myocardial damage by inducing the necroptosis route, while Se could act in the prevention of Cd-induced myocardial damage via the activation of the adiponectin pathway [149]. In addition, Se supplementation in laying chickens seems to change pancreatic ions and profile decreasing Cd accumulation in chicken pancreas [150].

Furthermore, enhanced incorporation of Sec after Se treatment led to increased TXNRD and GPX activities [151]. DNA repair and integrity can be affected by organic forms of Se. For instance, diets supplemented with SeMet at 3 or 6 μg/kg body weight per day for seven months were provided to elderly male dogs and the results were compared to untreated controls. Dogs supplemented with Se presented lower expansion of DNA damage in peripheral blood lymphocytes and in prostate cells compared to controls [152]. Moreover, supplementation of supranutritional organic Se to chicken nutrition, at safe subtoxic levels, prevented the peroxidation of health-promoting long-chain PUFA, like eicosapentaenoic acid (EPA) and DHA, and protected meat quality from oxidation [153]. Another study [154] revealed that SeMet stimulated cell protection against DNA damage and DNA repair. The increased repair complex formation in SeMet-treated cells was indicated as a feasible mechanism for the DNA repair response. Kibriya et al. [155] found that many genes, after Se supplementation, were upregulated. Further, Whanger et al. [156] stated that Se counteracts the neurotoxicity of Cd, Hg, V and Pb through a mechanism that induces their accumulation in brain, probably via a non-toxic complex. Selenium preventing action against DNA damage is affected by the used dose. For instance, a study on exocrine pancreas cells proposed a faster repair of DNA single-strand breaks in hamsters fed with 2.5 ppm Se than those fed with 0.1 ppm Se [157]. Selenomethionine could initiate the p53 tumor suppressor protein via a redox mode of action which needs the redox factor Ref1 [158]. Further, SeMet could protect against biological effects caused by radiation, via enhancement of DNA repair mechanisms in radiated cells. Consequently, SeMet may exert a particular action on the DNA repair machinery preventing DNA damage. Interestingly, this outcome is not the case or may be even opposite if selenite is used instead [159,160].

## 5. Actions and Properties of Selenoproteins

The mechanism of Se insertion into cysteine and further to selenoproteins has been extensively studied [161,162]. Similarly, many homologs of selenoproteins are not considered as such since they contain cysteine and not Sec [163]. Presently, the essentiality of Se is undoubted and its effects on the development of several diseases have been extensively reviewed in the literature [25,164,165]. Selenoproteins are essential for life and several selenoproteins have been characterized as antioxidant enzymes, protecting from damage caused by free radicals as already stated.

Functions of endoplasmic reticulum (ER) such as protein folding, post-translational modification, and transport are assisted by selenoproteins located in ER [166]. Further, the abundant 15 kDa SELENOF protein seems to control the quality of glycoprotein folding by improving glucosyltransferase activity of UDP-glucose: glycoprotein glucosyltransferase via formation of stable heterodimeric complexes [167]. Selenoprotein F may also function as oxidoreductase due to its thioredoxin-like folding which is mediated by CGU redox motif [168]. In addition, SELENOM, which shares a 31% sequence identity with SELENOF, enhances the formation of disulfide bond in proteins [168]. On the other hand, SELENOS and SELENOK participate in the machinery of the ER-associated protein degradation (ERAD) [166]. They are upregulated under conditions promoting protein misfolding. Furthermore, they are both single membrane-spanning parts of multi-protein complexes that transport misfolded proteins from the ER to the cytosol for degradation. Their exact way of action is elusive, though they probably act as chaperones for some ERAD substrates.

Identification of more than 50 selenoproteins has been performed since the discovery of Se incorporation in GPX [169]. Isolation and biochemical characterization of several of these proteins such as thioredoxin reductase, selenophosphate synthase, methionine-sulfoxide reductase and iodothyronine deiodinase has been achieved. Their significance for human health and physiology can be generally attributed to the special redox characteristics of the highly conserved active site Sec [170,171]. However, their mode of action is not known in detail. Localization of selenoproteins in various subcellular compartments is evidence of their critical function. For instance, almost one third of total human selenoproteins are located in the ER [166]. These include SELENOF, DIO2, SELENOM, SELENOK, SELENOS, SELENON and SELENOT. Type 2 deiodinase is important for thyroid hormone signaling [172] while redox regulation of calcium metabolism is connected with SELENOT and SELENON. Selenoprotein N is an in integral ER membrane protein that interacts with RyR an intracellular ryanodine receptor of Ca channel [173] and mutations that decrease the efficiency of selenocysteine-insertion lead to various muscle disorders.

## 6. Effects of Se Deficiency on mRNA Expression of Selenoproteins and Non-Selenoproteins

Selenium metabolism has been discussed in the literature with specific regard to the role of Se within the central nervous system [174]. There are also reviews on the action of Se on productive characteristics, antioxidative status, immune responses and nutrient utilization of poultry [175,176]. Members of this selenoproteome take part in diverse biological pathways and processes in chicken, such as in maintenance of the redox balance; interactions with metals and transport of Se. Six membrane-bound selenoproteins (selenoprotein I, selenoprotein K, selenoprotein S, selenoprotein T, DIO1 and DIO3) had pivotal roles in maintaining the membrane integrity. According to their ligand binding sites, chicken selenoproteins were classified as zinc-containing matrix metalloselenoproteins (selenoprotein 15, MSRB1, selenoprotein W and selenoprotein M), FAD-interacting selenoproteins (TXNRD1–3), prolyl oligopeptidase activity (POP)-containing selenoproteins (GPX1–4), secretory transport selenoproteins (GPX3 and selenoprotein Pa) and other selenoproteins [177].

The effects of Se deficiency on the induction of inflammation injury and oxidative damage to [178] chicken aorta vessels as well as on the deficiency of mRNA expression levels of selenoproteins, HSPs and immune functions in chicken have also been investigated. The results were linked with a downregulation of mRNA expression levels of 25 selenoproteins, upregulation of the mRNA expression of HSPs and immunosuppression [179,180]. Selenium deficiency can also lead to marked loss of selenoproteins activity, including GPXs, DIOs and TXNRDs [73].

The correlation of selenoproteins and Se deficiency causing apoptosis of chick embryonic vascular smooth muscle cells (VSMCs) has been examined [181]. Selenium deficiency led to higher cell apoptosis and decrease of cell viability. Selenium supplementation may mitigate these alterations. Moreover, at every level of Se supplementation, selenoprotein W1, selenoprotein 15, GPX1, GPX3, and GPX4 mRNAs were highly expressed in VSMCs [181]. An interesting work conducted in 55 days broilers with a Se-deficient diet (0.033 mg Se/kg) and a normal diet (0.2 mg/kg) showed that Se deficiency reduced HSP (HSP40, HSP60, and HSP90) and selenoprotein (selenoprotein K, GPX1, and selenoprotein H) mRNA expression, although the expression levels of selenoprotein Pb, selenoprotein I, selenoprotein N, MSRB1, HSP27, GPX2 and inflammatory factors (TNF-α, COX-2, HO-1 and iNOS) were upregulated. These results also indicated that selenoproteins play different roles in chicken testes [182]. Huang et al. [183] studied the effect of Se deficiency on selenoprotein expression levels in chicken muscular stomach and a correlation of muscular stomach injuries and selenoproteins was found. Selenium deficiency decreased the expression of 25 selenoprotein genes but raised the levels of mRNA expression of NF-κB, iNOS, TNF-α, COX-2, HO-1 and HSP27, HSP40, HSP60, HSP70 and HSP90. In other words, selenoproteins showed negative correlation with inflammation factors and HSPs. Therefore, the results showed that Se deficiency induced injuries in muscular stomach by reducing the levels of selenoproteins mRNA expression [183]. Selenium supplementation expedited and increased cell surface markers expression such as CD11c, CD40, CD86, and MHC II in a study investigating the impact of Se on chicken dendritic cells (DCs). Principal component analysis showed that the expression of selenoprotein W, selenoprotein K, DIO3, GPX1, GPX2, selenoprotein N, selenoprotein S, and selenoprotein H in chicken DCs was highly correlated, and selenoprotein W had highest correlation with the cell surface markers MHC II and CD11c [184]. Furthermore, Se-deficient, high energy diet in swine could cause oxidative stress, and downregulated the phagocytic dysfunction and the Nrf2 pathway in neutrophils.

The effect of Se deficiency on inflammatory factors and selenoprotein gene transcription in broiler kidneys was also studied. In the low Se diet group the expression of inflammation factors (TNF-α, COX-2, iNOS, and NF-κB) was raised, the mRNA expression level of 14 selenoprotein genes (GPX3, selenoprotein K, selenoprotein O, selenoprotein 15, selenoprotein H, selenoprotein P1, selenoprotein I, selenoprotein n1, selenoprotein W, selenoprotein T, selenoprotein U, selenoprotein S, DIO1 and DIO2) was reduced while the expression level of nine selenoprotein genes (selenoprotein M, selenoprotein SPS2, selenoprotein Pb, TXNRD1–3, and GPX1, GPX2, and GPX4) was elevated. The results indicated that Se deficiency caused changes in the expression of selenoprotein genes, kidney dysfunction and activation of the NF-κB pathway. In addition, Se supplementation can mitigate Pb toxicity effects via attenuation of apoptosis caused by Pb [185]. Finally, Se deficiency in chicken caused reduced tissue Se content as well as GPX and catalase activities, decreased the mRNA and protein expression of selenoprotein M and increased malondialdehyde (MDA) content in brain [186]. Under this contexts, recent transcriptional studies in chicken revealed that some selenoproteins, such as GPX4, are regulated at transcriptional level by high dietary Se. Supranutritional Se levels seem to down-regulate liver GPX4 mRNA levels, which means that reserves built by excess of Se may meet antioxidant requirements and no extra GPX4 transcription is required [187].

Other studies aimed to prove if chicken selenoprotein W has similar functions as mammal selenoprotein W and to examine its preventive action against H_2_O_2_ by affecting the expression of apoptosis and inflammation via developing a model of over-expressed or/and knocked down selenoprotein W in cultured chicken liver cells. Results revealed that after cell exposure to H_2_O_2_, levels of caspase-3, p53, Bak, Bax, prostaglandin E synthase (PTGEs, COX-2, TNF-α, NF-κB and iNOS were reduced in the selenoprotein W overexpressed cells but increased in the selenoprotein W knockdown cells in comparison to the H_2_O_2_-only treatment group. Levels of Bax, Bak, p53, COX-2, PTGEs, TNF-α, iNOS and NF-κB in the liver of Se-deficient chickens were highly increased compared to the control group [188]. Next, positive correlation has been reported in GPX1-4, DIO1, DIO3, selenoprotein S, selenoprotein N, selenoprotein T, selenoprotein W, selenoprotein 15, selenoprotein H, selenoprotein U and selenoprotein I with selenoprotein K, whereas selenoprotein P and DIO2 presented negative correlation with selenoprotein K. The above study was demonstrated by silencing selenoprotein K in chickens myoblasts [189].

In addition, studies have shown positive correlations between the mRNA expression of selenoprotein W and selenoprotein N genes and the quality (pH and muscle fiber diameter) of chicken muscle [190]. Cultured chicken liver cells exposed to H_2_O_2_ had lower levels of interleukin (IL)-1, IL-4, IL-6, IL-8, IL-10, IL-17 and interferon gamma (IFN-γ) in the selenoprotein W over-expressing cells but higher levels in the selenoprotein W knockdown cells compared to the H_2_O_2_ only control group. Besides, IL-1, IL-4, IL-6, IL-8, IL-10, IL-17 and IFN-γ in Se-deficient chicken serum and liver were much higher compared to the control group. Similar results have been shown in a study examining the effect of Se deficiency on the expression of selenoproteins and cytokines. The mRNA expression of 24 selenoproteins and seven cytokines (IL-2, IL-4, IL-8, IL-10, IL-12β, transforming growth factor (TGF)-β4, and IFN-γ) decreased, and the expression of three cytokines (IL-1γ, IL-6 and IL-7) increased in the Se-deficient group [191]. Selenoprotein W not only seemed to interfere with the destruction of H_2_O_2_ pathways, but also had a role in the immune system function. Studies have shown that inflammatory injury in immune tissues and cultured splenic lymphocytes can be influenced by Se in chicken, because it interacts with the expression levels of inflammatory factors (TNF-α, iNOS, NF-κB, COX-2 and PTGEs) and selenoprotein W. Further studies in selenoproteins’ gene expression and cytokine content in the chicken thymus have shown that, significant decrease in the expression levels of selenoproteins could result in oxidative stress in chicken thymus [192].

Studies with Chinese egg-laying ducks have shown that Se requirement for daily egg production is 0.18 mg Se/kg for early-laying birds, 0.24 mg Se/kg for peak-laying birds while for optimal GPX unction in peak-laying ducks it is 0.37 mg Se/kg [193]. Similarly, today’s minimum Se requirement in broilers is 0.15 μg Se/g diet and pancreas data indicate that the Se requirement should be raised to 0.2 μg Se/g diet [194]. Likewise, Turkey’s dietary Se requirement today is 0.2 μg Se/g and should be raised to 0.3 μg Se/g [195].

Concerns about the impact of inorganic and different organic Se sources on tissues and serum Se content, antioxidant capacity, performance, and liver mRNA expression of selenoproteins in broilers were also examined. The results suggested that Se sources did not affect broiler growth performance and organic Se exhibited highest Se concentration in serum, liver, and kidney [196]. Zhao et al. [197] examined the regulation of Se speciation, selenoproteins and degradation-related genes expression and Sec biosynthesis, by three forms of Se in broiler tissues. It has been shown that SeO further raised concentrations of total Se and SeMet in the liver, pectoral muscle, and thigh by 13–37% and 43–87%, respectively, compared with Se-yeast. Compared with inorganic Se or Se-yeast, SeO showed the ability to enrich SeMet and total Se accumulation, to induce the early mRNA expression of selenoprotein S and MSRB1, and to enhance protein expression of GPX4, selenoprotein P, and selenoprotein U in broiler tissues [197].

Molecular biomarkers in contrast to conventional ones seem to be better predictors of physiological effects connected with high Se intake. Investigations regarding super-nutritional Se effects on transcription did not identify well-regulated molecular biomarkers of high Se status. Microarray studies in rodents identified 14–242 genes altered by a Se intake of 1.0 μg Se/g as compared to Se deficient diets. From all these genes, the ones that were consistently regulated were the selenoprotein genes. Furthermore, the Se-specific effects noted were caused primarily by Se deficiency and not high Se [198].

## 7. Ranking in the Hierarchy of Selenoproteins

Selenoprotein synthesis is regulated [199,200,201,202] and a hierarchy in the expression of selenoproteins during Se deprivation and repletion has been reported [199]. The lowest ranking selenoprotein in the hierarchy is GPX1. Hierarchy of selenoproteins denotes that selenoproteins are not evenly supplied with Se, specifically not when Se gets limiting. Some selenoproteins disappear rapidly while others remain stable until deficiency becomes more serious. Therefore, rapidly disappearing selenoproteins, rank low in the hierarchy, while those remaining stable rank high. Instability is usually caused by the degradation of respective mRNAs [128].

It has been shown that some tissues and organs are more effective in maintaining Se levels and in synthesizing certain selenoproteins during Se deprivation compared to others. This is indicatory of differences in the biologic roles and in the selenoproteins requirements in different tissues [199,203]. The hierarchy during Se deprivation and repletion reveals the importance of particular selenoproteins and in turn determines the priority of the mRNA level and protein expression [50]. In Se-deficient states, the activities of most selenoproteins in the kidney, liver and lung decrease while in the brain remain at levels similar to those during normal Se intake levels [50]. Furthermore, stability of mRNA may be regulated by Se level since in Se deficiency cases excessive susceptibility to the nonsense-mediated decay pathway (NMD) and consequently mRNA decay has been observed [204,205,206].

Selenium may also efficiently control UGA-Sec codon translation [65,66], regulate levels of Sec tRNASer(Sec) and control the ratio of the methylated and unmethylated Sec tRNA(Ser)Sec isoforms [67,68,69,70]. The methylated isoform is translationally active and Se-induced tRNA methylation is a mechanism of Sel synthesis regulation. The methylated isoform has been shown to govern the synthesis of selenoproteins involved in the oxidative stress response such as GPX1 and GPX3, whereas the unmethylated form controls synthesis of housekeeping selenoproteins, i.e., TXNRD1 and TXNRD3 [71].

Selenoproteins’ hierarchy expression plays important role in Se metabolism into birds’ central nervous system (CNS). Brain is the last affected tissue by nutritional Se deficiency. However, selenoprotein expression follows a hierarchy by altering Se content so that GPX2-4, selenoprotein K, selenoprotein N, selenoprotein O, selenoprotein T, selenoprotein U, selenoprotein W, selenoprotein P1-2 and selenoprotein 15 take more necessary function in the CNS of chicken [207]. In contrast, Se deficiency down regulates chicken selenoprotein U mRNA levels in heart, kidney, liver, lung, muscle and spleen. On the other hand, selenoprotein U mRNA levels in testes and brain are not affected [208]. However, in Turkeys, studies on the expression levels of selenoproteins showed that expression of GPX1 was high in kidney, expression of GPX3 was high in all tissues except kidney, expression of selenoprotein U was high in liver, and selenoprotein W1 expression was high in heart, gizzard and muscle [209].

## 8. Recent Findings and Main Characteristics of Major Selenoproteins

Glycoprotein selenoprotein N (SELENON) is localized within the endoplasmatic reticulum (ER). Multiminicore disease (in the classical form) and the rigid spine muscular dystrophy are straight linked with SELENON [210]. Regulation of redox-related Ca homeostasis and antioxidant activity in cell is one of the major functions of SELENON [211]. Regulation of Ca levels in ER occurs through Ca pump ATP2A2 (ATPase sarcoplasmic/endoplasmic reticulum Ca^2+^ transporting protein 2) protection against the oxidative damage caused by endoplasmic reticulum oxidoreductase 1 alpha (ERO1A). Activity of ERO1A increases H_2_O_2_ concentration in the ER and this causes attack on ATP2A2 luminal thiols that leads to cysteinyl sulfenic acid formation (–SOH), which is reduced to free thiol (–SH) by SELENON, and in this way brings back ATP2A2 action [212]. In addition, it acts as RyR activity modulator by RyR oxidation prevention due to elevated oxidative stress and RyR redox state direct control through regulation of the RyR-mediated Ca mobilization which is required for normal differentiation and muscle development [173,213,214,215]. Further, SELENON is essential part of satellite cell maintenance and muscle regeneration [216]. Moreover, it seems that SELENON is necessary for Se regulation in mice uterine smooth muscle contraction [217]. On the other hand, intracellular Ca concentration rise is caused by overexpression of selenoprotein T [218]. Selenoprotein T plays a role in Ca homeostasis regulation and via cAMP-stimulating trophic factor in the control of the neuroendocrine secretion response [218]. Selenoprotein T is a protein with oxidoreductase activity as thioredoxin reductase and it is important in dopaminergic neurons protection against oxidative stress and apoptosis [219]. It is involved in neuroendocrine secretion, ADCYAP1/PACAP-induced Ca mobilization and has a role in redox regulation and fibroblast anchorage. In addition, contraction processes of the gastric smooth muscle are modulated by SELENOT which controls the release of Ca and myosin light chain kinase (MYLK) activation. In pancreatic islets, it contributes to ADCYAP1/PACAP-induced prolonged insulin secretion and is implicated in the glucose homeostasis control [219]. Moreover, a selenide-sulfide bond is maybe located between Cys-46 and Sec-49 in SELENOT polypeptide chain, which is considered to serve as redox-active pair. Further, the protective role of SELENOT and other selenoproteins on dopaminergic neurons against oxidative stress and apoptosis has been revealed recently. Selenoprotein T participates in neuron protection against oxidative stress and in the prevention of onset and intensive movement impairment in Parkinson Disease animal models [219].

A role in apoptosis and control of chemopreventive implications of Se seems to be attributed to SELENOF or also named selenoprotein 15 [73], which may be involved in redox reactions associated with the formation of disulfide bonds and in the quality control of protein folding in the ER. It is located in the ER and is expressed in higher levels in prostate and thyroid gland [220,221]. Selenoprotein M is distantly related to SELENOF but, its exact function is still elusive. Its role in causality of cancer and in early initiation of Alzheimer’s disease is under investigation. It has an oxidoreductase thiol-disulfide function which is involved in formation of disulfide bonds. By similarity, it is localized within the ER, but also within Golgi apparatus and perinuclear region [222].

Not only ER selenoproteins repair proteins. Methionine sulfoxide reductase B1 (MSRB1) is a selenoenzyme with wide distribution, which catalyzes the reduction of methionine (R)-sulfixide to methionine. Methionine oxidation is often a random effect induced by oxidative stress but it can also be a modification at post-translational stages which occurs on specific residue. Another MSRB1 function via methionine (R)-sulfoxide reduction is actin assembly and thereby stimulation of filament repolymerization. Besides, it contributes in innate immunity by reducing oxidized actin allowing repolymerization in macrophages. In vivo, immune response is controlled by MSRB1. Further, it promotes inflammatory cytokine gene expression in macrophages [223]. By similarity, to reverse random oxidation of methionine residues, MSRB1 exploits Sec [224] and, interestingly, it reduces more readily unfolded polypeptides than their folded counterparts, maybe due to better approach to otherwise hidden residues [225]. Therefore, cell protection from oxidative stress by methionine sulfoxide reductase enzymes can be explained by the recovery of unfolded proteins and oxidatively damaged nascent polypeptides. It has been recently shown that by regulating the oxidation state of two crucial methionine residues in actin monomer, MSRB1 controls mammalian actin assembly dynamics in collaboration with flavin-dependent monooxygenases (MICALs) [226]. Methionine stereoselective oxidation via MICALs causes disassembly of filaments and, on the other hand, reduction back to methionine via MSRB1 enhances filament assembly. Consequently, reversible sulfoxidation of methionine may serve as a general redox mechanism for protein folding/function regulation.

Selenoprotein K is essential for Ca flux in immune cells and in proliferation of T-cells and migration of neutrophils [166,167,168,227]. It is further implicated in ER-associated degradation (ERAD) of misfolded, soluble glycosylated proteins and Sec92 in SELENOK serves to stabilize the palmitoyl-DHHC6 intermediate by reducing hydrolyzation of the thioester bond until transfer of the palmitoyl group to the Cys residue on the target protein can occur [120]. Moreover, SELENOK is indispensable for CD36 cell surface expression and palmitoylation and contributes in foam cell formation and in low density lipoprotein (LDL) uptake by macrophages. In addition, selenoprotein K has been shown to protect cells from apoptosis induced by ER stress [121] and it preserves cells from oxidative stress when overexpressed in cardiomyocytes [122]. Further, a recent a study shows that the proliferation, migration, and invasion of human choriocarcinoma cells is mediated by SELENOK through negative regulation of human chorionic gonadotropin expression via p38 MAPK, Akt signaling and ERK pathways. In addition, SELENOK may act as suppressor of tumor in human choriocarcinoma cells by negatively regulating expression of β-human chorionic gonadotropin (β-hCG) via p38 MAPK, Akt and ERK signaling pathways [228].

Selenoprotein S contributes in the transfer of misfolded proteins from ER to the cytosol, for destruction by the proteasome in a ubiquitin-dependent manner. In addition, SELENOS is an efficient disulfide reductase [229]. SELENOS influences the activity and conformation of the ATPase complex VCP (vasolin-containing protein) which is involved in ubiquitination and translocation of misfolded proteins and DERL1 (degradation in endoplasmatic reticulum protein 1) which acts as a retro translocator into the cytosol [229]. Deprivation of glucose, together with tunicamycin and thapsigargin (stress inducers of ER), was shown to elevate gene and protein expression of SELENOS several-fold concomitantly with glucose-regulated protein 78. Moreover, SELENOS overexpression decreased Min6 cell oxidative stress-induced toxicity. These results indicate that SELENOS may be a member of the glucose-regulated protein family and a regulator of cellular redox balance [230]. Furthermore, the underling mechanism of SENELOS preventive role in atherosclerotic cardiovascular diseases was investigated [19,230]. Additionally, it may be implicated in the control of inflammation response [231]. The selenocysteine residue of SELENOS is localized in an essentially disordered region of the cytoplasmic C-terminal domain which interacts with VCP and DERL1 and indicates the formation of a DERL1-SELENOS membrane complex acting as VCP receptor. Furthermore, interaction has been shown with DERL2 and DERL3 as also with SELENOK gene which in turn interacts with VCP. In vitro studies showed that this residue provides high thioredoxin-dependent reductase activity [207,208] and that SELENOS may also act as a signaling molecule linking the unfolded protein response to the ERAD machinery

Eight homologs of GPX are found in mammals [232], five of which are selenoproteins, including GPXl (also known as cGPx), GPX2 (also known as GI-GPx), GPX3 (also known as pGPx), GPX4 (also known as PHGPx) and GPX6. More precisely, GPX family has a potent antioxidant function in cell cytosol (GPX1), gastrointestinal tract (GPX2), extracellular space and plasma (GPX3) and in cell membrane and sperm (GPX4). Glutathione peroxidase 5 is called epididymal GPX due to its exclusive localization in the epididymis [32]. Glutathione peroxidase 6, GPX7 and GPX8 where initially discovered via large-scale sequencing programs in mammals. The GPX6 is located in olfactory epithelium and in embryonic tissues [233,234]. Moreover, a study on β-cells in rats showed that antioxidant capacity of ER was improved by GPX7 or GPX8 without compromising production of insulin and the oxidative protein folding machinery [117].

As mentioned above, in the antioxidant system section, major functions of peroxidases are to remove and detoxificate H_2_O_2_ and hydroperoxides of lipids. Additionally, another important function is to preserve redox state of the cell. Apart from these, differentiation, signal transduction and pro-inflammatory cytokine synthesis regulation are also major physiological events where GPXs actively participate. In addition, GPXs have important roles in the antioxidant defense in spermiogenesis, in the maturation of spermatozoa and during embryonic development [235]. Glutathione peroxidases are involved in signaling cascades, e.g., GPX1 is involved in the molecular pathway of insulin signaling; GPX2 is involved in carcinogenesis; the membrane associated GPX3 has possibly peroxidative function in spite of low plasma concentrations of GSH; and GPX4 and GPX5 have major roles in apoptosis regulation, for full viability of mice and male fertility [236]. Though, the actions of GPX6 remain elusive [237]. However, applying SLIC (Sequence-and Ligation—Independent Cloning, a method for sequence-and ligation-independent cloning) [233] in a study on Huntington’s disease, it has been identified that toxicity of huntingtin mutation is modulated via age-regulated GPX6 gene. Moreover, in a mouse model, molecular and behavioral phenotypes linked with Huntington’s disease were drastically reduced after overexpression of GPX6 [234]. On the other hand, elucidation is awaited for GPX7 and GPX8 suggested involvement in protein folding [238,239]. The phospholipids-hydroperoxide glutathione peroxidase (GPX7 or NPGPx) which does not contain Sec residue is expressed in cancerous breast cells [240] and the name GPX8 has been proposed for a novel member of the GPX family, the existence of which was revealed via phylogenetic analysis in amphibians and mammals [241].

Three protein types with a variety of specific roles in thyroid metabolism are included in the iodothyronine deiodinase family. These 3 deiodinases (DIO1–3) are found in all vertebrates and have notable roles in activating thyroxine (T4) and inactivating both T3 and T4. Their importance resides in the fact that T4 must be activated by deiodination to the short-lived biologically active T3 for thyroid hormone action initiation [242]. Bioactive 3,5,3′-tri-iodothyronine (T3) is converted to thyroxin (T4) by DIO1 and DIO2. Thioredoxin reductases, deiodinases and glutathione peroxidases are all present in the thyroid gland and contribute in biosynthesis of thyroid hormone, redox control of thyrocytes, antioxidant defense and in the metabolism of thyroid hormone [243].

Other selenoproteins that may not be part of a family include, but are not restricted to, the selenophosphate synthetase 2 (SEPHS2), SELENOI, SELENOH, SELENOO, SELENOP, SELENOW, and SELENOV. Moreover, in eukaryotes, the selenoprotein family includes the Fep15, SELENOJ, SELENOU1, the plasmodium selenoprotein, SELENO1–SELENO4 and the protein disulfide isomerase (PDI) which is narrowly distributed in eukaryotes. While the role of some of them is still largely unknown, the role of others is clearly understood. The greater attention has been received by the selenoproteins in humans. More specifically, SEPHS2 is involved in selenophosphate formation intended for selenoprotein synthesis [244] and forms selenophosphate from selenide and ATP. Furthermore, it is located in the cytosol and binds ATP, nucleotides and Se.

Expression level regulation in de novo GSH synthesis relative genes and response to redox status phase II detoxification is regulated by SELENOH [245]. Overexpression of SELENOH gene in hippocampal neuronal cells has neuroprotective effects against ultra violet B (UVB)-induced cell death. In addition, SELENOH overexpression is involved in mitochondrial biogenesis and mitochondrial functional performance [246].

Selenoprotein I in *E. coli* catalyzes biosynthesis of phosphatidylethanolamine from cytidine diphosphate (CDP)-ethanolamine and it has a main role in vesicular membranes formation and maintenance. Selenoprotein I seems to be involved in the formation of phosphatidylethanolamine via “Kennedy” pathway (catalytic activity) [247,248]. However, until now, the function of selenoprotein I is largely elusive.

Selenoprotein O is a protein with wide distribution that has homologs in yeast, bacteria, animals and plants, although its function is still undiscovered [249,250]. It has high priority for Se supply and this is deduced by the slight Se effect on selenoprotein O gene expression. It may be a redox-active mitochondrial selenoprotein which interacts with a redox target protein.

Selenoprotein P is a glycoprotein with a high Sec content, which is abundant in extracellular environment. Its functions include endothelium located antioxidant defense, Se transport among tissues and Se homeostasis [251]. Additionally, it is the main selenoprotein in plasma and contains at least 40% of the whole amount of Se in plasma [252]. Moreover, SELENOP plasma level is a finer indicator of nutritional Se compared to the previously used GPX3 [73] because a greater Se intake is required for SELENOP than GPX3 full expression [253].

Selenoprotein V is about 37 kDa and is mainly produced in chicken testes. This selenoprotein contains specific amino acid sequence motives that predict a role in redox-regulation, although its exact function is unexplained [22]. On the other hand, selenoprotein W seems to be implicated in antioxidant protection of cardiac and skeletal muscle, protecting chicken embryonic myoblast cells against H_2_O_2_ mediated apoptosis. Selenoprotein W regulates also inflammation-related cytokines during H_2_O_2_ chicken liver damage [254,255]. Finally, regarding selenoprotein D, a recent study has been conducted using the TargeTron gene knockout system to explore the role of selenoproteins in *Clostridium difficile* nosocomial pathogen [256]. TargeTron insertion into selenoprotein D resulted in a significant growth deficiency and global loss of Se incorporation. Stickland metabolism could be a potential target for antibiotic therapies in the future.

## 9. Conclusions

Selenoproteins need for their synthesis several cofactors and depend mainly on Se intake through the diet. The organism pays a high cost of energy for the production and preservation of these cofactors for selenoprotein synthesis which consequently suggests the significance of this family of proteins to cell function. Notwithstanding, the functions of selenoproteins are fairly heterogeneous. Selenoproteins play essential roles in numerous conditions and diseases including neurodegeneration and endocrine disorders, cardiovascular disorders and cancer. On account of the various functions of selenoproteins, strategies targeting function and/or expression of specific selenoproteins could be considered for prevention and therapeutic treatment of disorders. Different forms of dietary Se may selectively increase synthesis of specific selenoproteins. Pharmaceuticals could also target factors involved in selenoprotein synthesis or specific selenoproteins. Nevertheless, the functions of many selenoproteins are still elusive, which is why understanding the function of each member of the selenoprotein protein family will be ssignificant for defining the health benefits of Se.

## Figures and Tables

**Table 1:** Reactive oxygen and nitrogen species.

Radicals	Non-Radicals
RO^•^, Alkoxyl	H_2_O_2_, Hydrogen peroxide
HOO^•^, Hydroperoxyl	HOCl, Hypochlorous acid
HO^•^, Hydroxyl	O_3_, Ozone
ROO^•^, Peroxyl	^1^O_2_, Singlet oxygen
O_2_^•^¯, Superoxide	ONOO¯, Peroxynitrite
NO^•^, Nitric oxide	NO¯, Nitroxyl anion
NO_2_^•^, Nitrogen dioxide	HNO_2_, Nitrous acid
